# Role of Canagliflozin on function of CD34+ve endothelial progenitor cells (EPC) in patients with type 2 diabetes

**DOI:** 10.1186/s12933-021-01235-4

**Published:** 2021-02-13

**Authors:** Seshagiri Rao Nandula, Nabanita Kundu, Hassan B. Awal, Beda Brichacek, Mona Fakhri, Nikhila Aimalla, Adrian Elzarki, Richard L. Amdur, Sabyasachi Sen

**Affiliations:** 1 The GW Medical Faculty Associates, Washington, DC USA; 2 Department of Medicine, The George Washington University, 2300 Eye Street, SMHS, Room 462,, Washington, DC 20037 USA; 3 Department of Medicine and Endocrinology, Veterans Affairs Medical Center, Washington, DC USA

**Keywords:** Canagliflozin, Type 2 diabetes, Progenitor cells, Endothelial function, Urinary exosomes

## Abstract

**Background:**

Endothelial progenitor cells (EPCs) has been shown to be dysfunctional in both type 2 diabetes mellitus (T2DM) and chronic kidney disease (CKD) leading to poor regeneration of endothelium and renal perfusion. EPCs have been shown to be a robust cardiovascular disease (CVD) risk indicator. Effect of sodium glucose channel inhibitors (SGLT2i) such as Canagliflozin (CG) on a cellular biomarker such as CD34+ve progenitor cells, which may help predict CVD risk, in patients with T2DM with established CKD has not been explored.

**Methods:**

This is a pilot study where 29 subjects taking metformin and/or Insulin were enrolled in a 16 week, double blind, randomized placebo matched trial, with a low dose 100 mg CG as the intervention group compared to matched placebo. Type 2 diabetes subjects (30–70 years old), with hemoglobin A1c (HbA1c) of 7–10%, were enrolled. CD34+ve cell number, migratory function, gene expression along with vascular parameters such as arterial stiffness, serum biochemistry pertaining to cardio-metabolic health, resting energy expenditure and body composition were measured. Data were collected at week 0, 8 and 16. A mixed model regression analysis was done and p value less than 0.05 was considered statistically significant.

**Results:**

A significant expression of CXCR4 receptor with a concomittant increase in migratory function of CD34+ve cells was observed in CG treated group as compared to placebo group. Gene expression analysis of CD34+ve cells showed an increase in expression of antioxidants (superoxide dismutase 2 or SOD2, Catalase and Glutathione Peroxidase or GPX) and notable endothelial markers (PECAM1, VEGF-A, and NOS3). A significant reduction in glucose and HbA1c levels were observed along with improved systolic and diastolic blood pressure in the CG group. A significant increase in adiponectin (p = 0.006) was also noted in treatment group. Urinary exosomal protein leak in urine, examining podocyte health (podocalyxin, Wilm’s tumor and nephrin) showed reduction with CG

**Conclusion:**

Low dose Canagliflozin has a beneficial effect on CD34+ cell function, serum biochemistry and urinary podocyte specific exosomes in type 2 diabetes.

## Background

Diabetes mellitus is a major public health concern worldwide as number of patients with type 2 diabetes mellitus (T2DM) is increasing rapidly and it is gradually reaching the proportions of a world epidemic. Currently, 463 million adults are living with diabetes and by 2045 estimated number of adult living with diabetes will reach to 700 million [1, 2]. Centers for Disease Control and Prevention (CDC) reported approximately, 9.4% of American adults have diabetes [1, 2]. Diabetes is closely associated with oxidative stress, inflammation, and as a result endothelial dysfunction and cardiovascular complications [1–3]. On the other hand, 15% of US adults are estimated to have chronic kidney disease and 38% of end stage renal disease developed Chronic kidney disease (CKD) as a consequence of diabetes [4]. Use of a sodium-glucose linked transporter inhibitor (SGLT-2i) has shown promise in improving glycemic control, weight reduction, hypertension and even changes in circulating renin–angiotensin–aldosterone system (RAAS) and nitric oxide (NO) [5, 6]. However, whether these group of drugs have any effect on cardiovascular disease (CVD) risk modification or specifically on endothelial cell or endothelial progenitor cells, is unclear. It is extremely important to evaluate improvement, especially cardiovascular and endothelial health of the patient after administration of diabetic drugs.

Available therapeutics are focused on glycemic control to delay the diabetes related complications [7, 8]. Widely used oral drugs to control hyperglycemia in patient with diabetes are metformin, dipeptidyl peptidase 4 (DPP4) inhibitors, and SGLT2 inhibitors. Several studies showed SGLT2 inhibitor can be beneficial compared to DPP4 inhibitors, especially on subjects with type 2 diabetes and mild chronic kidney disease. A use of a SGLT-2 inhibitor has a promise to improve glycemic control, obesity and hypertension as reported by several research groups [9–13]. Meta analysis studies have shown less risk of heart failure with SGLT2 inhibitors in comparison with DPP4 inhibitor and Glucagon-like peptide-1 (GLP1) agonist [14]. However, recently several studies have shown canagliflozin (CG) specifically lower the risk of cardiovascular disease in pre-clinical and clinical studies in type 2 diabetes [15–21]. Recent studies suggested CG may inhibit endothelial pro-inflammatory chemokine/cytokine secretion through 5′ adenosine monophosphate-activated protein kinase (AMPK) dependent and independent mechanisms [22] and CG even helps to improve myocardial ischemia–reperfusion injury [23].

Serum based biomarkers are being used for long time to identify and monitor cardiovascular improvement. However, use of serum based inflammatory markers are inefficient and does not change until the endothelium is already damaged or inflamed and produce paracrine pro-inflammatory molecules which subsequently gets detected [24]. For this reason serum based biomarkers are unable to appropriately detect effect on endothelium at an early stage. Several studies recently has shown that endothelial progenitor cells (defined as CD34+ve cell) can serve as an important biomarker to monitor cardiovascular function [25–29].

Endothelial progenitor cells (EPC) defined as CD34+ve progenitor cells are obtained from the hematopoietic stem cell pool by magnetic bead column separation (MACS Miltenyi Biotech). EPCs contribute to vascular repair, angiogenesis and also vasculogenesis [28–31].

It is well established that EPCs respond to a chemo-attractant such as stromal derived factor 1 alpha (SDF1α). The EPCs migrate towards a chemoattractant such as SDF1a, released by wounded/damaged/ apoptosis prone tissue [30, 31] This process of targetted migration of EPCs has been described as “homing-in” [26]. The recruitment of EPCs help to repair the damaged tissue. It is also known that EPCs release or secrete growth factors and cytokines that promote regeneration as part of their paracrine properties [32, 33]. We reported earlier that CD34+ve cells help to monitor effect of a therapy or intervention in an early onset [26, 28, 29]. A few preclinical and clinical studies showed, improvement of cardiovascular health by using a combination of DPP-4 inhibitor and metformin in type 2 diabetic patient by improving endothelial dysfunction and vascular complications from diabetes by increasing number of CD34+ve EPCs and migratory capacity [29, 34]. There are also some previous studies that indicate that SGLT2 inhibitors may help to improve endothelial dysfunction both in patients and animal studies [35–40], however such studies don’t give us much information at a cellular level.

Previous studies from our laboratory have shown that CD34+ve cells, derived from peripheral blood can act as a cellular biomarker that is more reliable than serum based markers for CVD risk estimation [26, 28, 29]. Arterial stiffness assays also help define endothelial function independent of serum assays but corelates well to standard biochemical assays that are used to define endothelial function [41, 42]. In our previous studies using two di-peptylpeptidase inhibitors (DPP4i) such as saxagliptin and linagliptin in type 2 diabetes mellitus (T2DM) patients [43, 44] we have demonstrated that CD34+ve cells are responsive to a change in therapy or intervention within 2–4 weeks using CD34+ as a biomarker and CD34+ cell based assays can be used as a reliable non serum based cellular bio-marker. The same is true for of a prediabetes population with an aerobic exercise intervention, where changes in gene expression are quite fast [29]. CD34+ve cells or endothelial progenitor cells have been used clinically to improve collateral circulation and have been extensively studied as a robust cardiovascular biomarker. Therefore, studying CD34+ve cells in T2DM subjects with or without Canagliflozin can give vital information about the medication and its effect on hematopoetic stem cell pool that may have a long-term effect on future endothelium and other vascular cells. Our non cellular outcome measures were focused on parameters that are agreed to be indicators of endothelial function such as arterial stiffness, serum inflammatory markers and markers of renal podocyte health.

In this 16-week intervention study we investigated the synergistic effect of canagliflozin, a SGLT2 inhibitor and metformin, on number and function of CD34+ve EPCs while adjusting for variables that may affect stem cell number and function such as hemoglobin A1c (HbA1C), body mass index (BMI), gender and age.

## Methods

### Trial design and oversight

This is a phase 4 (post-marketing), two arm, single site, parallel group, double blind, placebo controlled randomized clinical trial comparing low dose Canagliflozin 100 mg tablets, taken orally, once daily, with matching placebo. The study was conducted in accordance with Good Clinical Practice guidelines set forth by the International Conference of harmonization and any local regulatory guidelines with the approval and oversight of the George Washington University Institutional Review Board. The trial was funded by Janssen Scientific Affairs, LLC and conducted by the Investigator-Sponsor Dr. Sabyasachi Sen at the George Washington University.

Subjects were initially pre-screened to assess eligibility. Once determined preliminary eligibility, they were brought in for a screening visit to confirm eligibility via interview, medical record check and laboratory workup once the subject signed the informed consent. The subjects (n = 29) were then enrolled into one of two arms of the study: 100 mg Canagliflozin or matching placebo. 15 subjects were enrolled into the active canagliflozin group and 14 subjects were enrolled into the placebo group.

There were 3 study visits total, first at week 0, second at week 8 (mid-point) and third and last visit at week 16. All three of the visits had same assessments. The assessments that were done were: vital measurements, adverse event (AE) check and a peripheral blood draw. Approximately 80 ml of blood was drawn for CD34+ve endothelial progenitor cell harvesting and routine blood work.

Other parameters tested were resting metabolic rate (RMR, energy expenditure), measurement of waist to hip ratio, urine sample collection, Tanita body composition scale, pulse wave analysis and pulse wave velocity to determine arterial stiffness. Subjects were advised to adhere to 150 min of weekly aerobic exercise and their activity levels were monitored using ACTi graph activity monitor.

A follow up phone call visit was done 30 days from the last in-person visit to assess for any residual adverse events (AE).

### Participants

Subjects were included if they were between 30 and 70 years old inclusive, with a diagnosis of Type 2 Diabetes for 15 years or less. HbA1c inclusions were between 7.0 and 10.0% inclusive. Their baseline medications were stable dose of Insulin (either short acting or long acting) and/or Metformin (1–2 g/day). A stable dose was considered to be at least the maximum labeled dose or dose not associated with unacceptable side effects. Patients with BMI between 25 and 39.9 kg/m^2^ were included, thereby excluding severe obesity. Only, patients with impaired renal function were included, with Chronic Kidney Disease stage 1 to 3, with lowest eGFR cut-off of 30 ml/min/1.73 (GFR, as calculated by MDRD formula). During the duration of the study alterations in baseline medications were done to keep HbA1C between 7 and 8% across all subjects.

Any patients with Type I diabetes, history of Diabetic ketoacidosis, low hematocrit (less than 28 units), history of recent pancreatitis or cancer, recent coronary or cerebrovascular event within 6 months, use of consistent steroid medications, untreated thyroid disease were excluded.

Additional Inclusion and Exclusion criteria can be found in Appendix.

### Outcome objectives

The primary objective is to ascertain if addition of Canagliflozin improves CD34+ve cell number, (CD34+ve number, %CD34+ve of total Mononuclear Cell population) function (cell migration function in response to SDF1α) and gene expression, in Type 2 Diabetes Mellitus, which will be correlated to improvement in 24 h urinary protein estimation and serum Creatinine Clearance.

The secondary objective is to correlate the cellular outcome measures with other measures of endothelial function such as Arterial Stiffness [measured by pulse wave analysis (Augmentation Index) and pulse wave velocity (m/s)], Serum Biochemistry (CMP, IL6, hsCRP, Leptin, Serum insulin, TNFα), Adiposity (as % body fat), resting energy expenditure (in kcal) and Glycemic control (through HbA1c).

### Body composition measurement

Body composition was measuring using Tanita™ BF-350 Body Composition Scale and manually. Manual measurement included height, waist circumference, hip circumference. Tanita scale (Tanita Corporation of America, Inc, USA) uses a bio-impedance electrical impulse to measure body fat percent, fat mass (kg), fat free mass (kg), percent body water, water mass (kg) alongside weight. It then calculates the BMI and estimated basal metabolic rate.

### Basal metabolic rate measurement

Resting Emergency Expenditure (REE) was measured using KORR REEVUE (Korr Medical Technologies, USA). Test was conducted with the subject sitting and well rested. Subject was instructed to keep a tight seal around the mouthpiece and use the nose clip to avoid breathing in from the nose. The test ran for about 10 min. It calculated estimated REE, predicted REE, estimated Total Energy Expenditure (TEE), VO_2_ Max and estimated calorie intake per day.

### Arterial stiffness

This parameter was measured using Atcor Sphygmocor CP system (Atcor Technologies, USA). We obtained two outcomes such as: pulse wave velocity and pulse wave analysis. The patient was supine on the examination table, three leads were attached on right forearm, left forearm and left shin.

Pulse wave analysis (PWA) was measured on the left Radial Artery with the subject supine. At least three readings were taken with Operator Index ≥ 80. Measurement includes Augmentation Index (AI), Augmentation Index adjusted for Heart Rate of 75 (AI-75), Augmentation Pressure (AP), Aortic and Radial reading of systolic, diastolic, pulse pressure and mean pressure.

Pulse wave velocity (PWV) was measured with the subject supine. This measurement requires a distal and proximal artery. Distal was used as right femoral artery with proximal being the left carotid. Index and ring fingers were used to manually localize the pulse, sometimes an arterial Doppler was used to localize the femoral pulse on patient with challenging body habitus. Once a stable pulse waveform was observed, the probe position was kept stable for 20 more pulses before the reading was finalized. Three readings were taken with standard deviation of less than 10%. The result reported a velocity in m/s, alongside the standard deviation with error.

### Biological sample and vital collection

A venous blood sample was collected from the Antecubital fossa. About 80 ml of blood was collected. 60 ml for EPC analysis and 20 ml for standard of care blood works which included Basic Metabolic Panel, Lipid Panel, HbA1c, hsCRP, IL6, Adiponectin and Insulin. Enzyme linked immune assay (ELISA) was performed to analyze serum GLP1 and SDF1α using ELISA Immunoassay kit (Raybiotech, Norcross, GA) for GLP1 and Sandwich ELISA (EHCXCL12A, Thermo Scientific, USA) for SDF1α. Urine sample was collected for urine Microalbumin and Creatinine ratio. Vitals were gathered on the left arm, Systolic Pressure, Diastolic Pressure and heart Rate, along with sublingual temperature. Serum nicotinamide adenine dinucleotide (NAD/NADH) were measured by using NAD/NADH assay kit from Abcam, MA, USA (Catalog No. ab65348) and Ketone bodies were from serum were measured by using Ketone Body Assay Kit from Millipore Sigma, MO, USA (Catalog No. MAK134).

### ACTi graph activity monitor

Subjects level of activity was measured using Actigraph wGT3x-BT activity monitors (ActiGraph Inc, USA). Subjects were advised on diet and exercise instructed to wear the meter during all waking hours and was advised to adhere to 150 min of moderate intensity aerobic exercise per week. Actigraph served as a measure of this exercise compliance, and to verify for exercise as a confounding variable.

### Polyethylene glycol (PEG) enrichment of extracellular vesicles

The cells debris and large apoptotic bodies were removed from the urine samples by centrifugation at 500×*g* for 5 min followed by 3000×*g* for 30 min at 4 °C. Transfer supernatant into ultracentrifugation tubes and centrifuged at 100,000×*g* at 4 °C for 75 min (Optimal XPN-100 centrifuge, Beckmann Coulter Inc, USA). After ultracentrifugation the pellet was dissolved in RIPA buffer with protease inhibitor cocktail and stored the sample at − 80 °C for further analysis.

### Extracellular vesicle characterization and Western blotting

Extracellular vesicles were isolated by ultracentrifugation and identified by the expression of CD9, CD81, CD63 AND HSP70 markers in western blot. Extracellular vesicle extracts were fractionated by SDS-PAGE and transferred to a polyvinylidene difluoride membrane using a transfer apparatus according to the manufacturer’s protocols (Bio-Rad, USA). After incubation with 5% nonfat milk in TBST (Bio-Rad, USA) (10 mM Tris, pH 8.0, 150 mM NaCl, 0.5% Tween 20) for 60 min. The membrane was washed once with TBST and incubated with antibodies against CD9 (1:1000), CD81 (1:1000), CD63 (1:1000), HSP70 (1:1000) (Sysetem Biosciences, USA), anti-podocalyxin (PODXL, 1:1000), anti-wilms tumor protein (1:1000) and anti-Nephrin antibody (1:1000) at 4 °C for 12 h (Abcam, USA). Membranes were washed three times for 10 min and incubated with a 1:20,000 dilution of horseradish peroxidase-conjugated goat anti-rabbit antibody for 90 min at room temperature. Blots were washed with TBST three times and developed with Pierce ECL kit (ThemoFisher Scientific, USA).

### Cellular and clinical assessments

#### CD34+ve endothelial progenitor cell analysis

Peripheral blood samples (approximately 60 ml) were drawn from patients and phosphate buffered saline (1:1) was added. Identification and quantification of circulating cell phenotypes was performed on fresh blood samples, within 3 h after collection, using flow cytometry. Briefly, mononuclear cells (MNCs) were then isolated from whole blood using a Ficoll density centrifuge method. MNCs were counted and aliquot was used for CFU-Hill colony formation assay following the manufacturer’s instruction (Stem Cell Technologies, Vancouver, BC, Canada). Colony forming unit was counted at day14. A fraction of the MNC were stained with fluorescein isothiocyanate (FITC)-conjugated antihuman CD34, Allophycocyanin (APC) conjugated antihuman CD184 (CXCR4) and FITC conjugated antihumanCD31 antibodies (Miltenyi Biotec GmbH, Bergisch-Gladback, Germany) in order to analyze specific progenitor cell surface marker (CD34) and mature endothelial cell surface markers (CD31) or receptor for SDF1a ligand, CXCR4) by flow cytometry. After gating mononuclear cells in the side scatter (SSC)-A vs forward scatter (FSC)-A plot, CD34/CD184 single- and double-positive cells were identified. Cells were acquired on a fluorescence-activated cell sorter (FACS) Canto instrument (Becton Dickinson, USA) and scored with the FloJo software.

To isolate EPCs (CD34+ve), MNCs were magnetically sorted through a column after cells were stained with CD34+ve microbeads antibody (Miltenyi Biotec GmbH, Bergisch Gladback, Germany). An aliquot of CD34+ve cells were then stained with trypan blue and counted using an Auto Cellometer Mini (Nexcelom Bioscience, USA) to assess viability.

CD34+ve gene expression analysis was performed by quantitative reverse transcriptase polymerase chain reaction (qRT-PCR) as previously described [33]. CD34+ve cell total mRNA was extracted and purified using the RNeasy Minikit (Qiagen, Germany). mRNA was then converted into cDNA by using the high capacity cDNA reverse transcriptase kit (Thermo Fisher Scientific, MA) Possible gene expression changes promoted by Canagliflozin was assessed by a CFX96 real-time PCR systems (Bio-Rad, CA) using Taqman Universal masters Mix II (Thermo Fisher Scientific, USA) and inventoried probes. The gene expression analysis included antioxidants, apoptosis, endothelial functions, chemotaxis, inflammation and endothelial lineage cell surface markers. The expression of each individual gene was normalized to either housekeeping 18S or GAPDH and calculated using C-ddct method considering the difference in cycle threshold between visit 2 and 3 and baseline (visit 1).

The migratory capacity of CD34+ve was evaluated using the CytoSelect 24-well Cell Migration Assay kit (Cell Biolads, Inc., San Diego, CA). Cells were suspended in Serum free media and seeded at 100,000 cells per insert. Migration of the cells through a 3 µm polycarbonate membrane to the wells containing a serum-free media (control) and chemoattractant SDF-1α (10 or 100 ng/ml) (from Sigma-Aldrich, USA) was assessed after cells were kept overnight in incubator. Migratory cells were dissociated from the membrane and subsequently lysed and quantified by fluorescence (480 nm/530 nm) using CyQuant GR dye (Cells Biolabs, Inc, USA). The fluorescence ratios between cells exposed to the chemotactic factor and cells exposed to chemoattractant-free media (control) along the visits were used to analyze the migratory capacity of the cells.

### Statistical analysis

Continuous variable distributions were examined for skewness or outliers using histograms. When these were present, we log-transformed the variables. Outliers > 5SD from the mean were capped at 5SD from the mean. We used 2-tailed between-groups t-tests to examine differences between treatment groups at baseline on continuous variables, and either chi-square or the Fishers Exact test for categorical variables. To examine differences between treatment groups across all time points, as well as time effects, and whether the slope of change over time differed between treatments, we used random effects mixed model regression, examining the main effects of treatment (Canagliflozin vs. placebo), and time (v1, v2, v3), and the treatment by time interaction. This method allows us to use all non-missing subject data and adjusts for within-subject auto-correlation. For variables with significant effects in the mixed models, we examined the means graphically. SAS (version 9.4, Cary, NC) was used for data analysis with p < 0.05 considered significant.

Since subjects were randomized to treatment, chance of baseline subject characterizations acting as confounders are minimized. Therefore, randomized control trials do not usually adjust for baseline differences. However, in small studies, imbalances may exist as a result of random group assignment, and may thus function as mediators of an association between treatment and outcome. In this situation, one would need to adjust for the mediators in order to obtain the direct effect of treatment on outcome, which is the effect of interest.

## Results

### Primary outcome

The study population was representative of subjects with uncontrolled type 2 diabetes, but with no preexisting macro-vascular complications. All adverse effects that occurred throughout the duration of the study were either not related to the study medication and design or fell within the expected side effects profile for Canagliflozin. Table 1 shows blood biochemistry measures across the three visits. No statistical significance was observed between the groups for body composition measures. Mean percent body fat is low and mean percent body water is high in canagliflozin group and showed no significance difference throughout the study (Table 2). This implies that the changes that we note in cellular and serum based outcome measures are independent of body composition changes.

**Table 1:** Blood biochemistry before and after Canagliflozin treatment

Parameter	Placebo (n = 14)			Canagliflozin (n = 15)			Fixed effects test P value
	Visit 1	Visit 2	Visit 3	Visit 1	Visit 2	Visit 3	
eGFR	85.22 ± 4.43	79.85 ± 4.22	88.33 ± 4.52	80.24 ± 4.27	81.13 ± 4.56	79.90 ± 4.21	0.31
Creatinine	0.84 ± 0.05	0.94 ± 0.05	0.80 ± 0.06	0.96 ± 0.05	0.88 ± 0.06	0.95 ± 0.05	0.37
HDL	51.56 ± 3.60	52.23 ± 3.66	51.67 ± 3.69	42.96 ± 3.47	46.97 ± 3.48	46.17 ± 3.48	0.28
Cholesterol	163.40 ± 10.09	166.23 ± 10.53	147.76 ± 10.61	167.78 ± 9.74	176.26 ± 9.84	171.57 ± 9.84	0.20
LDL/HDL	1.68 ± 0.15	2.26 ± 0.15	1.60 ± 0.16	2.19 ± 0.15	1.43 ± 0.16	2.08 ± 0.15	0.92
C-reactive protein	1.03 ± 0.2	0.40 ± 0.2	0.92 ± 0.2	0.54 ± 0.2	0.96 ± 0.2	0.63 ± 0.2	0.44
Insulin	2.49 ± 0.21	2.63 ± 0.20	2.71 ± 0.22	2.50 ± 0.20	2.46 ± 0.22	2.56 ± 0.20	0.42
Augmentation Index (AI)	22.14 ± 2.9	24.58 ± 3.1	21.37 ± 3.2	24.2 ± 2.8	23.21 ± 2.9	28.67 ± 2.8	0.17
PWV	11.19 ± 0.64	10.85 ± 0.68	11.42 ± 0.70	9.59 ± 0.66	9.70 ± 0.65	10.12 ± 0.63	0.86
Leptin	39.84 ± 3.34	42.42 ± 3.52	40.28 ± 3.65	28.36 ± 3.30	29.92 ± 3.36	27.79 ± 3.29	0.96
SDF-1α	6.34 ± 0.47	5.74 ± 0.45	5.85 ± 0.47	5.49 ± 0.46	6.41 ± 0.48	5.68 ± 0.44	0.51
3Hydroxy-butyric acid	0.03 ± 0.01	0.0	0.03 ± 0.01	0.01 ± 0.01	0.001 ± 0.01	0.02 ± 0.01	0.75
Acetoacetic acid	0.72 ± 0.25	0.0	0.30 ± 0.27	1.08 ± 0.24	0.05 ± 0.25	0.69 ± 0.24	0.86

**Table 2:** Cellular parameters before and after Canagliflozin treatment

Parameter	Placebo (n = 14)			Canagliflozin (n = 15)			Fixed effects test P value
	Visit 1	Visit 2	Visit 3	Visit 1	Visit 2	Visit 3	
Mean MNC	167.00 ± 16.08	153.79 ± 16.08	155.09 ± 16.08	147.75 ± 15.54	145.93 ± 15.54	155.09 ± 15.54	0.71
Mean CD34+ve	2527.64 ± 508.69	3128.79 ± 508.69	3157.36 ± 508.69	2452.07 ± 491.44	2031.40 ± 491.44	2682.93 ± 491.44	0.32
% of CD34+ve	1.01 ± 0.19	1.04 ± 0.17	1.23 ± 0.21	0.94 ± 0.19	0.85 ± 0.22	0.93 ± 0.17	0.75
% of CD31+ve	2.85 ± 0.33	2.47 ± 0.44	2.16 ± 0.43	2.75 ± 0.33	2.34 ± 0.38	2.58 ± 0.35	0.59
CD34+CD184	0.57 ± 0.09	0.45 ± 0.10	0.61 ± 0.11	0.43 ± 0.09	0.33 ± 0.10	0.37 ± 0.09	0.62
CFU	10.30 ± 2.03	13.62 ± 2.10	13.61 ± 2.19	6.08 ± 1.95	7.62 ± 1.95	7.53 ± 1.89	0.82
SDF 10 NG	0.20 ± 0.031	0.20 ± 0.031	0.23 ± 0.03	0.15 ± 0.03	0.15 ± 0.03	0.26 ± 0.03	0.26
SOD2	0.97 ± 0.23	1.45 ± 0.23	0.92 ± 0.24	1.00 ± 0.22	1.05 ± 0.24	1.36 ± 0.24	0.22
Catalase	− 0.03 ± 0.25	− 0.09 ± 0.25	− 0.61 ± 0.26	− 0.55 ± 0.23	− 0.55 ± 0.26	0.20 ± 0.26	0.04
GPX35	2.00 ± 0.54	2.00 ± 0.54	1.71 ± 0.54	2.00 ± 0.47	1.64 ± 0.50	2.25 ± 0.54	0.68
CXCL12	1.00 ± 0.16	0.53 ± 0.17	0.33 ± 0.17	1.00 ± 0.15	0.60 ± 0.16	0.63 ± 0.18	0.59
CXCR4	1.06 ± 0.17	1.03 ± 0.17	0.90 ± 0.18	1.00 ± 0.16	1.03 ± 0.18	1.58 ± 0.18	0.06
EDN1	1.22 ± 0.27	1.05 ± 0.29	1.03 ± 0.29	1.00 ± 0.24	0.79 ± 0.27	2.10 ± 0.27	0.02
VEGEF-A	0.95 ± 0.17	1.46 ± 0.18	0.83 ± 0.19	1.00 ± 0.17	0.86 ± 0.18	1.12 ± 0.19	0.04
PECAM	0.94 ± 0.11	1.43 ± 0.11	0.89 ± 0.12	1.00 ± 0.11	0.87 ± 0.12	1.28 ± 0.12	0.002
KDR	1.00 ± 0.18	0.53 ± 0.18	0.50 ± 0.20	1.00 ± 0.18	0.55 ± 0.20	1.16 ± 0.20	0.13
NOS3	1.11 ± 0.21	0.06 ± 0.21	− 0.25 ± 0.21	0 ± 0.18	− 0.23 ± 0.19	0.34 ± 0.21	0.08
Wilm’s tumor	6.52 ± 0.65	6.13 ± 0.72	6.36 ± 0.75	6.27 ± 0.60	6.68 ± 0.62	5.75 ± 0.59	0.45
Nephrin	6.15 ± 0.55	5.10 ± 0.62	5.87 ± 0.65	5.39 ± 0.51	5.70 ± 0.53	5.24 ± 0.50	0.25
PODXL	6.72 ± 0.33	6.08 ± 0.38	6.68 ± 0.40	7.05 ± 0.31	6.92 ± 0.32	6.62 ± 0.30	0.34

### Venous blood biochemistries

Venous blood biochemistries were gathered, both through Labcorp of America and through serum ELISA and both standard of care and research labs were collected.

**Fig. 1:**
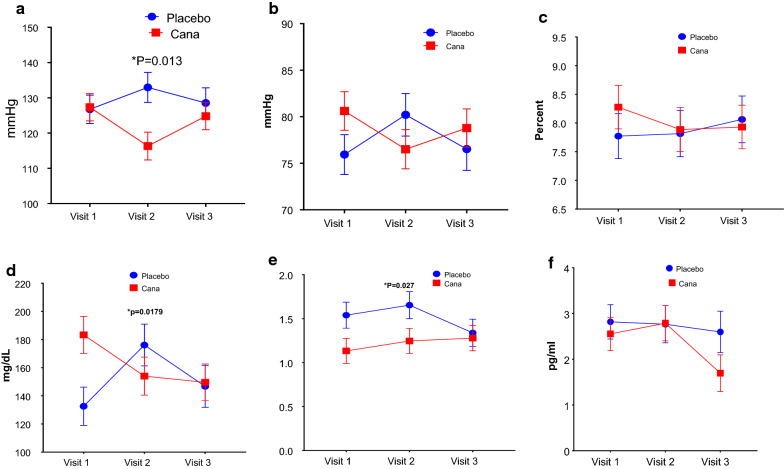
A significant difference in mean systolic blood pressure (**a**) and diastolic blood pressure (**b**) is observed between Canagliflozin and placebo groups. In Canagliflozin group both decreased from visit 1 to 2 and increased by visit 3. Whereas in placebo group both increased from visit 1 to 2 and decreased by visit 3. **c** HbA1c went down substantially in Canagliflozin group whereas it increased in Placebo group. The sharp decrease in HbA1c is seen in visit 1to visit 2 (p = 0.0919) and is stabilized from visit 2 to visit 3 in Canagliflozin group. **d** Glucose levels also followed the same trend as HbA1c in Canagliflozin group by sharp decrease from visit 1 to 2 and then stabilized till visit 3. Whereas, in placebo group glucose levels increased from visit 1 to 3. **e** A significant rise in adiponectin levels is noted in Canagliflozin group where as a sharp decrease is observed in placebo group from visit 2 to 3. **f** Inflammatory marker serum IL6 levels decreased. The difference was significant from visit 2 to 3 (p = 0.0259) in Canagliflozin group as compared to placebo group

Detailed lab values of selected significant parameter are on Table 1. We found statistically significant decrease in the Glucose levels in Canagliflozin group (p = 0.01), whereas an opposite effect is seen in placebo group. A sharp decrease in HbA1c levels from visit 1 to 2 in Canagliflozin group (p = 0.0919) and overall decrease in HbA1c as compared to placebo group (Fig. 1) exert a beneficial effects on endothelial progenitor cell function. The inflammatory marker IL-6 levels in serum decreased significantly. As shown in the Fig. 1, in Canagliflozin treated subjects from visit 1 to visit 3 IL-6 levels reduced (P = 0.05). Adiponectin and endocrine factor synthesized and released from adipose tissue. We have observed a significant increase in adiponectin levels (p = 0.02) in Canagliflozin group, where as in placebo group adiponectin levels decreased from visit 1 to 3 (Fig. 1).

To study the effect of Canagliflozin on mitochondrial function serum NAD and NADH levels in placebo and Canagliflozin groups, we measured serum NAD and NADH levels from visit 3. We did not see any significant difference between the groups (for Placebo 0.8454 ± 0.3239 and for Canagliflozin 0.7335 ± 0.2730). Serum ketone bodies, 3hydroxybutyric acid and acetoacetic acid were measured. As shown in the Table 1, there is no significant difference between the groups between either of the two ketone bodies.

### Arterial stiffness

Stiffness of an artery significantly contributes to lack of pliability and contractility and is an important marker of increased peripheral resistance, diastolic dysfunction and systemic hypertension. It is associated with cardiovascular diseases in older individuals and is positively associated with hypertension, Coronary Artery Disease, stroke, heart failure and atrial fibrillation [41, 42]. Arterial stiffness is assessed using parameters such as AI adjusted for a heart rate of 75 (AI-75) and PWV. The mean PWV for canagliflozin group started at a lower level as compared to placebo group and is not significantly different from visit 1 to 3. In the placebo group PWV is high at visit 1 and remains high until visit 3 (Table2).

Even though there is no significant difference in augmentation index AI [adjusted for a heart rate of 75 (AI-75)] and PWV between placebo and Canagliflozin treated subjects, there was a significant reduction in mean systolic blood pressure (p = 0.01), noted in Canagliflozin group as compared to Placebo group. Similarly significant decrease in mean diastolic blood pressure (p = 0.02) was noted in Canagliflozin group as compared to placebo group.

### Adiposity

Body composition measurement showed no statistically significant change amongst the group throughout the visits. As expected, given short duration of the treatment the subjects were asked to maintain activity level as advised by American Diabetic Association (ADA) for healthy living. There was no statistically significant change in hip to waist ratio, body weight and body fat percent amongst the treatment and control group.

### Cellular outcome measures

#### Characterization of endothelial progenitor cells (CD34+ve)

To study the effect of SGLT2 inhibitor, Canagliflozin, on the endothelial progenitor cell number we counted the total CD34+ve cells by using Cellometer Mini (Nexcelom Biosciences, Lawrence, MA), after microbead column separation (BD Biosciences, San Jose, CA). As shown in Table 2, there is no statistical significance in difference in CD34+ve cell numbers between placebo and Canagliflozin group.

To discern the direct effect of Canagliflozin on CD34+ve cells percentage the values were adjusted for HbA1c. HbA1c adjusted value for CD34+ve cell number is statically significant (p = 0.0047). Consistent with this, HbA1C adjusted values for dual positive cells, CD34+ve/CD184+ve cells, were also statistically significant (p = 0.0039) between the groups, with higher number in Canagliflozin group.

#### CFU‑Hill’s colonies

The CFU-Hill’s colony formation was improved in the Canagliflozin treatment group. CFU numbers increased sharply from visit 1 to 2 and visit 1 to 3 (data not shown). HbA1c adjusted value for CFU is statically significant (p = 0.042) with higher CFU count in canagliflozin group. In placebo group despite an increase in CFU number from visit 1 to 2 there was an overall decrease in CFU number from visit 1 to 3.

#### Migration response

**Fig. 2:**
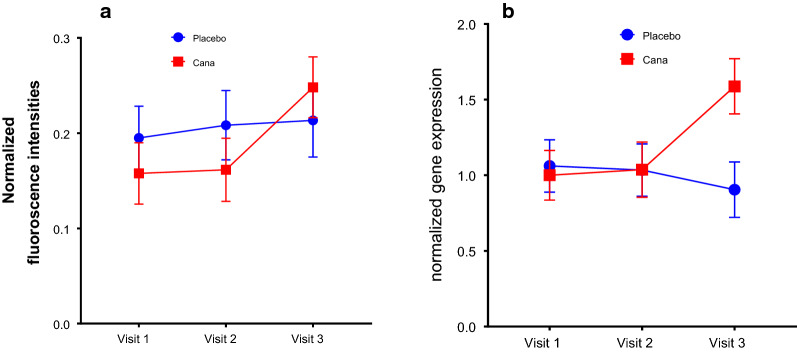
Migration Assay in response to SDF1alpha at 10 ng/ml concentration and gene expression of SDF1a receptor, CXCR-4 on CD34+ve cells. **a** Mean migration of CD34+ve cells towards SDF1-α (at 10 ng/ml) increased from visit 1 to 3 in Canagliflozin group as compared to placebo group (for visit 1 to 3 p = 0.03 and for visit 2–3 p = 0.05). **b** CXCR4 gene expression on CD34 positive cells is increased (p = 0.06) in Canagliflozin group as compared to placebo group at visit 3

Next, we wanted to study the effect of Canagliflozin on migration of CD34+ve cells, using Bowden’s chanber. The migratory response of CD34+ve cells to the chemotactic factor SDF1α (concentration of 10 ng/ml) increased in canagliflozin group as compared to placebo group. However from visit 2 to 3 there was a significant increase in migration of CD34+ve cells in canagliflozin group as compared to placebo group (Fig. 2).

### Gene expression analysis

#### The effect of Canagliflozin on the gene expression of CD34+ve cells

**Fig. 3:**
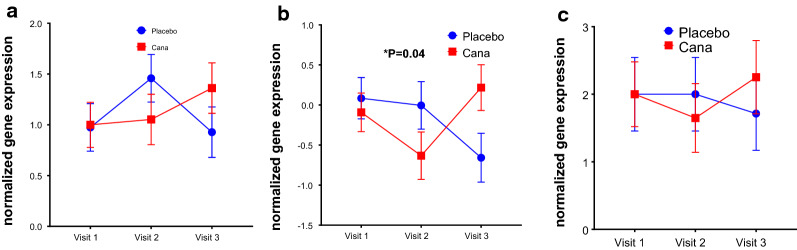
The effect of Canagliflozin on CD34+ve cell antioxidants gene expression. Fold change in gene expression of antioxidants SOD2 (**a**) in CD34+ve cells are increased in Canagliflozin group from visit 1 to 3. However, the same for the placebo group is decreased. Whereas the antioxidants, **b** Catalase (CAT) and **c** GPX3 gene expression increased in CD34+ve cells in Canagliflozin group from visit 2 to 3. However, the same for the placebo group is decreased. Overall, all the antioxidant genes showed increased expression going from visit 2 to visit 3, whereas the placebo there was a downward trend with catalase expression reaching statistical difference, overall

**Fig. 4:**
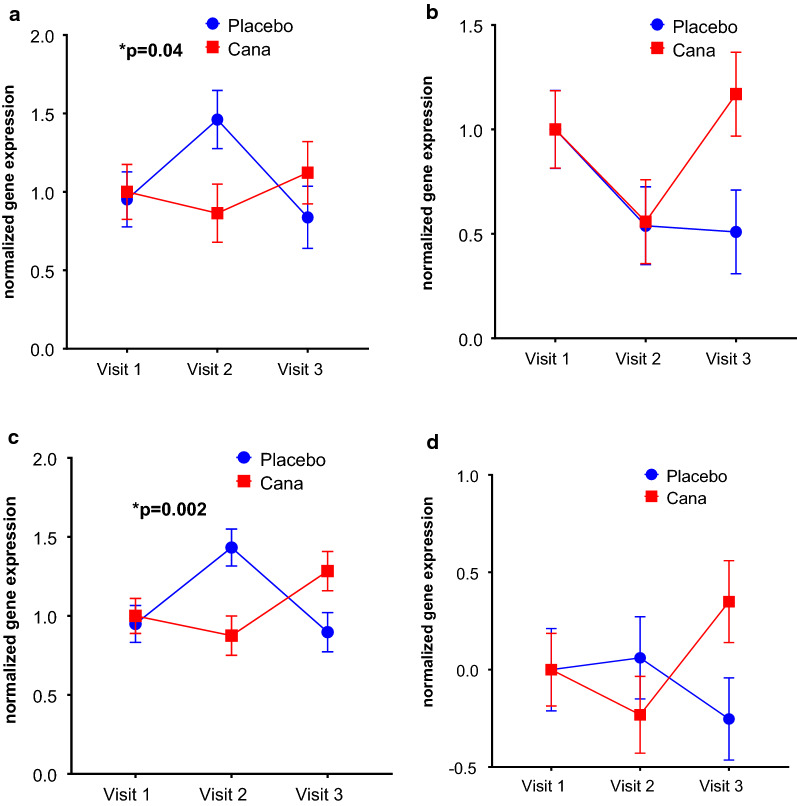
The effect of Canagliflozin on CD34+ve cell Endothelial markers gene expression. Fold change in gene expression of Endothelial markers **a** VEGF-A, **b** KDR and **c** PECAM and endothelial function marker **d** NOS3 on CD34+ve cells increased significantly in Canagliflozin group from visit 1 to 3. However, the same for the placebo group is decreased. Gene expression is normalized to 18S

We wanted to study the gene expression on CD34+ve cells for antioxidants SOD2 (superoxide dismutase 2), GPX3 (glutathione peroxidase 3), CAT (catalase) genes. The mean fold change in gene expression of all 3 antioxidants (Sod2, p = 0.22, CAT, p = 0.04, GPX3, p = 0.68) that we studied were increased in canagliflozin group where as in placebo group it is decreased from visit 1 to visit 3 (Fig. 3a–c). The mean gene expression for endothelial markers VEGF-A (p = 0.04), KDR (p = 0.13) and PECAM1 (p = 0.02) had increased significantly in Canagliflozin group from visit 1 to visit 3 (Fig. 4a–c) as compared to placebo group. Since the prominent endothelial gene expression (VEGF) is increased significantly, we were interested to see the expression of endothelial functional genes NOS3(endothelial nitric oxide synthase). Over all there is a trend in increase in NOS3 expression (P = 0.08) in Canagliflozin treated group as compared to placebo group though no statistical significant difference was observed between the groups (Fig. 4d). We also checked the inflammatory markers and we did not see any differences in the inflammatory markers IL6 and TNF-α expression between the groups (data not shown), however the serum IL-6 levels decreased in Canagliflozin treated group as compared to placebo group (Fig. 1f). This is difference is statistically significant from visit 2 to visit 3 (p = 0.0259).

### Urine podocyte function marker

#### Quantification of exosomal proteins in urine samples by western blot

Our recent study using Linagliptin showed protection from renal injury with patients taking Linagliptin for a duration of 12 weeks [44]. However the difference between Linagliptin and placebo was not picked up by urine albumin: creatine ratio but was noted on assessing urinary exosome podocyte markers. We were interested to study the effect of Canagliflozin on podocyte health by quantifying the exosome expression for Nephrin, Wilm’s Tumor (WT-1) and podocalaxyn like protein 1 (PODXL) in urine samples from placebo and Canagliflozin group. These three proteins are specific for podocyte health.

**Fig. 5:**
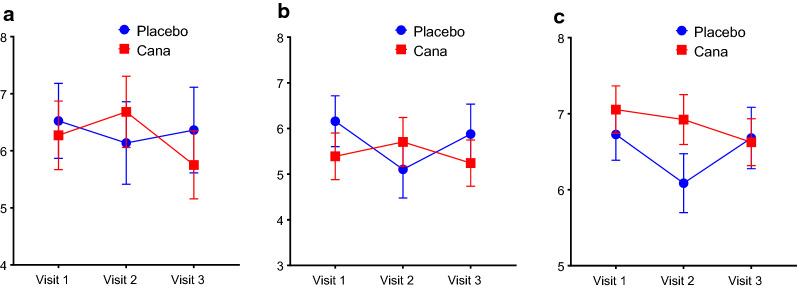
Urinary exosome markers **a** Wilm’s tumor-1 (WT-1), **b** Nephrin and **c** Podocalyxin (PODXL) were identified by Western blot and followed by quantification. A trend in reduced mean band intensities for Urinary exosome markers are observed in Canagliflozin group from visit 1 to visit 3 as compared to placebo group

As described in the methods after isolating exosomes from urine samples of both placebo and canagliflozin group exosomes were identified as CD9 positive. As shown in the Fig. 5, the mean band intensities for the podocyte specific exosomal protein leak of PODXL and Nephrin and WT-1 were decreased from visit 1 to 3 in Canagliflozin group as compared to placebo group. Even though the difference in band intensities between the groups is not significant (taking all visits together) a trend in decrease intensities is observed in Canagliflozin treated group, particularly in visit 2 to visit 3, indicating a late onset protection effect of Canagliflozin. Interestingly, the HbA1c adjusted value for Nephrin in Canagliflozin treated group was statically significant (p = 0.0133).

## Discussion

### Primary outcome: cellular

Reduction in CD34+ve cells is a consistent observation in people with T2D and this defect may contribute to the development of chronic micro and macrovascular complications. Previous studies demonstrated that SGLT2 inhibitors reduce the risk of cardiovascular events in patients with T2D and high cardiovascular risk [45, 46]. Despite several attempts to explain such striking cardiovascular protection exerted by SGLT2 inhibitors we still have a poor understanding of the potential mechanisms at work. Therefore, in this study we attempted to test whether SGLT2 inhibitor Canagliflozin exert beneficial effects on endothelial progenitor cells which are considered as biomarkers and have a role in the pathogenesis of chronic diabetic complications. We investigated the effect of SGLT2 inhibitor Canagliflozin in addition to metformin and/or Insulin on CD34+ve EPCs and CD34+ve CD184+ve cells as a marker for vascular endothelial function. Our data shows canagliflozin did not increase the endothelial progenitor cells in this short time 16 week study. CD34+ve cells decreased from visit 1 to 2 and then increased to slightly higher by the end of Visit 3 in canagliflozin group. The decrease in EPCs is in parallel with the recent study on effect of SGLT2 inhibitors on EPCs [36]. Even though, CD34+ve cells are not increased, in number, statistically, the expression of CXCR4 and SDF1 (CD184+ve) expression are increased in the CD34+ve cells. This finding is correlated with the increased migratory response to SDF1-α of CD34+ve cells in canagliflozin group (Fig. 2b). It is likely that long term treatment with canagliflozin will increase the function of CD34+ve cells along with good glycemic control [46].

Next, in order to understand the effect of Canagliflozin on gene expression of CD34+ve cells we performed qPCR analysis. We looked at pathways involving inflammation, oxidative stress, EPC chemotaxis pathways and endothelial function markers, in CD34+ve cells obtained post magnetic column separation of MNCs. We found the expression of SDF1-α receptor CXCR4 expression is very close to statistical significance in Canagliflozin treatment. This was an unexpected result as we did not expect an SGLT2i to have effect of stem cell migration, unlike DPP4i treatment where migration can be expected to increase secondary to increased SDF1a increased bio-availability. Antioxidants and endothelial markers are upregulated in Canagliflozin treatment group, indicating improved vascular health. The upregulation of endothelial markers, VEGF-A and its receptor KDR indicates VEGF signaling most likely plays an important role in vasculogenesis and tissue repair upon Canagliflozin treatment. Interestingly eNOS expression increased significantly from visit 2 to visit 3 in Canagliflozin group. Another interesting findings were increase in serum adiponectin levels by ELISA and reduction in serum IL-6 values particularly between visit 2 to visit 3. These two results concurrently indicate improved endothelial health following canagliflozin treatment (Fig. 1e, f).

As nephropathy is an important progressive complication of T2DM we specifically looked at markers beyond urine microalbumin: creatinine ratio which did not show a difference between the groups. However, when we examined a more sensitive marker such as urine exosomes that specifically look at podocyte derived protein such as podocalcyxin, nephrin and Wilm’s tumor we noted that canagliflozin appears to reduce these podocyte proteins particularly between visit 2 to 3.

Lastly, we wanted to examine any possible differences in ketone bodies between placebo and low dose canagliflozin as the latter has been shown to cause euglycemic ketosis [47], however at this dose we did not note any difference in serum ketone body levels. There are reports of canagliflozin influencing mitochondrial function via AMPK-Sirt1-Pgc-1α signalling pathway [48] however we will note any differences in NAD: NADH ratio, however it will be worthwhile to investigate into these effects at higher doses.

## Limitations of our study

Limitations of our pilot study may include the relatively short duration of 16-week Canagliflozin therapy, which may have been inadequate to see significant changes in certain clinical and cellular parameters. This may have been also because of the small sample size. However, we were satisfied to note statistical significance in various cellular outcomes and even in some serum based markers. Further studies with a larger population and longer duration may be helpful to further define the mechanisms behind our findings.

## Conclusion

Canagliflozin when added to subjects with Type 2 Diabetes along with metformin and/or Insulin, demonstrates a functional improvement of CD34+ Endothelial Progenitor Cell migratory function through increased CD34/CXCR4 positivity. Canagliflozin also promotes improvement in blood pressure, arterial stiffness and serum biochemical markers that indicates improved endothelial function.

We believe CD34+ cells can act as a valuable biomarker for assessment of endothelial function, in a setting of diabetes and can help provide valuable clinical information leading to appropriate therapeutic intervention choices. It may also allow an individualistic approach to diabetes medication choice rather than standard serum based assays with set normal ranges.

## Data Availability

All associated data will be available to the public, as requested. Demography of subjects including detailed description of baseline characteristics and parameters have been included in the Appendices.

## Inclusion criteria

1. Adults aged 30–70 years.
2. Diagnosis of type 2 diabetes within the previous 15 years using criteria of the American Diabetes Association.
3. Currently treated with a stable dose of Insulin, Metformin (1–2 g/day), or a stable combination of the two as therapy.
4. HbA1C between 7 and 10% (both inclusive).
5. BMI 25 to 39.9 kg/m^2^ (both inclusive).

## Exclusion criteria

1. Implanted devices (e.g., pacemakers) that may interact with Tanita scale.
2. Previous coronary or cerebrovascular event within 6 months of screening or active or clinically significant coronary and/or peripheral vascular disease.
3. Low hematocrit (< 28 units).
4. Pre-existing liver disease and/or ALT and AST > 2.5X’s UNL.
5. CKD stage 4 and,
6. History of pancreatitis, or cancer (except basal cell carcinoma and cancer that is cured or not active or being treated in the past 5 years).
7. Statin use started or dose change in the last 3 months.
8. Use of oral anti-diabetic medication other than Metformin, or Insulin.
9. Use of consistent long-term steroid medication in the last 3 months (oral, inhaled, injected).
10. Systolic BP > 140 mmHg and Diastolic BP > 90 mmHg.
11. Active wounds or recent surgery within 3 months.
12. Inflammatory disease, or chronic current use of anti-inflammatory drugs within the last 3 months.
13. Triglycerides > 450 mg/dl.
14. Untreated hyper/hypothyroidism.
15. Auto antibody confirmed type 1 diabetes.

Additionally, patients who are active smokers, patients who are pregnant, nursing women, and post-menopausal women who are on hormone replacement therapy will be excluded.

Patients on low dose oral contraceptives will be allowed to participate as these formulations contain lesser amount of estrogens.

## Abbreviations

AI: Augmentation Index
AI-75: Augmentation Index adjusted for a heart rate of 75
AP: Augmentation Pressure
AS: Arterial stiffness
BCl-2: B-cell lymphoma 2
BMI: Body mass index
BUN: Blood urea nitrogen
CAT: Catalase
CDKN1A: Cyclin-dependent kinase inhibitor 1A
CD34: Progenitor marker
CFU: Colony forming units
CVD: Cardiovascular disease
CXCR4: C-X-C Motif Chemokine Receptor 4
DBP: Diastolic blood pressure
DPP-4 inhibitors: Dipeptidyl peptidase-4 inhibitors
EDN-1: Endothelin 1
eGFR: Estimated glomerular filtration rate
ELISA: Enzyme-Linked Immunosorbent Assay
eNOS: Nitric oxide synthase 3 (endothelial cell)
EPCs: Endothelial progenitor cells
FFM: Fat free mass
GAPDH: Glyceraldehyde-3-phosphate dehydrogenase
GLP1: Glucagon like peptide 1
GPX1: Glutathione peroxidase 1
HbA1C: Hemoglobin A1C, glycated hemoglobin test
IGF1: Insulin-like growth factor 1
IL-6: Interleukin 6
kg: Kilograms (weight)
lbs: Pounds (weight)
MNCs: Mononuclear cells
PECAM1: Platelet and endothelial cell adhesion molecule 1
PWA: Pulse wave analysis
PWV: Pulse wave velocity
qRT-PCR: Quantitative reverse transcriptase polymerase chain reaction
REE: Resting energy expenditure
SBP: Systolic blood pressure
SDF1α: Stromal cell-derived factor-1α
SOD1, SOD2: Super oxide dismutase 1 & 2
TBW: Total body water
TNFα: Tumor Necrosis Factor α
TP53: Tumor protein p53
VEGF: Vascular endothelial growth factor
VEGFA: Vascular endothelial growth factor A
VEGFR2: Vascular endothelial growth factor receptor 2
VO2: Maximal oxygen consumption
18S: *18S* Ribosomal RNA
